# Legalon® SIL: The Antidote of Choice in Patients with Acute Hepatotoxicity from Amatoxin Poisoning

**DOI:** 10.2174/138920112802273353

**Published:** 2012-08

**Authors:** Ulrich Mengs, Ralf -Torsten Pohl, Todd Mitchell

**Affiliations:** a Research & Development, Rottapharm | Madaus, Madaus GmbH, 51011 Cologne, Germany; b Dept. of Family Medicine, Dominican Hospital, Santa Cruz, CA 95076, USA

**Keywords:** *Amanita phalloides* poisoning, Amatoxin, Acute Hepatic Failure, Antidote, Legalon® SIL, Silibinin.

## Abstract

More than 90% of all fatal mushroom poisonings worldwide are due to amatoxin containing species that grow abundantly in Europe, South Asia, and the Indian subcontinent. Many cases have also been reported in North America. Initial symptoms of abdominal cramps, vomiting, and a severe cholera-like diarrhea generally do not manifest until at least six to eight hours following ingestion and can be followed by renal and hepatic failure. Outcomes range from complete recovery to fulminant organ failure and death which can sometimes be averted by liver transplant. There are no controlled clinical studies available due to ethical reasons, but uncontrolled trials and case reports describe successful treatment with intravenous silibinin (Legalon® SIL). In nearly 1,500 documented cases, the overall mortality in patients treated with Legalon® SIL is less than 10% in comparison to more than 20% when using penicillin or a combination of silibinin and penicillin. Silibinin, a proven antioxidative and anti-inflammatory acting flavonolignan isolated from milk thistle extracts, has been shown to interact with specific hepatic transport proteins blocking cellular amatoxin re-uptake and thus interrupting enterohepatic circulation of the toxin. The addition of intravenous silibinin to aggressive intravenous fluid management serves to arrest and allow reversal of the manifestation of fulminant hepatic failure, even in severely poisoned patients. These findings together with the available clinical experience justify the use of silibinin as Legalon® SIL in *Amanita *poisoning cases.

## 1 INTRODUCTION

Human poisoning with cytotoxic mushrooms (*Amanita phalloides* and related species) is associated with severe morbidity and a high mortality rate due to rapidly progressive fulminant hepatic failure. 

While fatal mushroom poisoning has long been recognized as a major health problem in Europe, the gathering and consumption of wild mushrooms is popular in some immigrant populations as well as mushroom enthusiasts in the US leading to an increasing health hazard [1, 2]. Despite warnings, edible and toxic mushrooms such as *Amanita phalloides* are frequently mistaken by mushroom collectors. In rare cases, *Amanita* mushrooms have been ingested in suicide attempts [3].

Epidemiological data on mushroom poisoning, particularly poisoning with amatoxin-containing mushrooms, are rare. The true incidence of this intoxication is unknown since national data banks maintained at poison control centers have only incomplete data of this non-reportable illness [4].

Human mushroom poisonings are classified according to clinical symptoms. In the case of intoxication with *Amanita* species - the most relevant being *Amanita phalloides* - the summary of typical clinical symptoms is called *phalloides syndrome*. While severe mushroom poisonings are rare, *phalloides syndrome* is reported to account for about 90% of fatal mushroom poisonings [5].

(Table **1**) lists the *Amanita* species responsible for the great majority of all fatal mushroom poisonings found throughout Europe and increasingly in North America [5, 2]. 

It is estimated that there are 100 to 200 fatalities per year due to mushroom poisoning in the United States and Europe [6]. Ingestions and deaths from other amatoxin containing *genera* like *Lepiota* and *Galerina* have also been reported [7, 8].

Prior to the 1980s the mortality rate following amatoxin ingestion was reported to be higher than 50%. Over the past 25 years the mortality rate has been reduced to 10-20% primarily due to improved supportive and intensive care [2].

## 2 HEPATOTOXICITY OF AMATOXIN 

Two different classes of toxins, amatoxins and phallotoxins can be distinguished in most of these potentially fatal mushrooms. Another class of toxins, virotoxins, appears to be restricted to *Amanita virosa* [5].

The phallotoxins, present in all *Amanita* species, have a high affinity for intracellular F-actin muscle filaments, thus blocking their polymerization reaction. Since they are not absorbed via the enteral route, phallotoxins are thought to play no role in human intoxication [9, 10]. Toxicity in the *phalloides syndrome* is due to amatoxin which is well absorbed, extremely thermostable, water-soluble, and resistant to acids and enzymes [5, 9].

Amatoxin refers to the amanitins, a family of bicyclic octapeptides (molecular weight approx. 900 daltons). The most relevant of which, α- and β-amanitin, account for more than 90% of the amatoxin content in *Amanita phalloides.* [11].

The amatoxin concentration in *Amanita phalloides* has been determined to be 0.2 to 0.4 mg per gram of fresh tissue. The human lethal dose is 0.1 mg/kg body weight. Ingestion of 5 to 7 mg of amatoxins, equivalent to less than 50 g of fresh mushrooms, may be lethal [5].

### 2.1 Amatoxin Kinetics 

From kinetic investigations in dogs and in humans, it is known that amatoxin is readily absorbed through the intestinal epithelium, does not bind albumin, and disappears rapidly from the plasma [12, 13].

About 60% of absorbed amatoxin is excreted into bile and undergoes enterohepatic circulation [14]. Consequently, the exposure of the liver cells to the toxins is prolonged. Kinetic studies in humans have shown that amatoxin is detectable in plasma at low concentrations during the 24 to 48 hour period after ingestion. No correlation could be found between amatoxin plasma concentrations and the clinical severity or outcome after intoxication [13]. The toxin is excreted in the urine and may be detectable up to 3 to 4 days after ingestion. Efforts are underway to develop a clinically useful rapid diagnostic kit [9].

### 2.2 Mechanism of Amatoxin Toxicity

Amatoxin reaches the liver cell by sinusoidal transport systems which are responsible for the recycling of bile salts under physiological conditions. Penetration into liver parenchymal cells is rapid [6, 15].

Experimentally it has been shown that amatoxin binds 1:1 with a subunit of the DNA-dependent RNA polymerase II, which is responsible for the transcription of DNA to mRNA. The formation of mRNA is inhibited, protein synthesis disrupted, and necrosis of the liver ensues with some latency due to the slow degradation of the mRNA reservoir [16]. 

Cells with high rates of protein synthesis are most vulnerable and lead to the initial gastrointestinal symptoms as well as the later development of hepatic and renal failure. Microscopic examination of the hepatic parenchyma after intoxication reveals fatty degeneration, as well as an abnormal concentration of lipids and carbohydrates in the cell nuclei with a pattern of centrilobular necrosis and hemorrhage [1]. In the case of the liver, hepatocytes are damaged early, while the hepatic sinusoids are spared. For the hepatocytes it is assumed that amatoxin concentrations as low as 3 x 10 ^-7^ M can block 90 % of the transcription activity for mRNA. This critical toxin concentration may be reached within 1 hour after ingestion of large amounts of amatoxins [2, 9].

In the kidney, cellular damage to the proximal tubules and irreversible lesions in the late phase of intoxication were observed with low doses of amatoxins in animal experiments [9, 17].

After being exposed to amatoxin, cells suffer from a deficiency of transcriptional information leading to a dearth of essential proteins and finally to cell death. The crucial factor for cell survival is the period of time that amatoxins are present inside the cell at a concentration sufficient to inhibit the transcription process (≥ 10^-8^ M), which is in turn dependent on the concentration of amatoxin in the extracellular medium [9].

A promising therapeutic approach will lower the amatoxin concentration at the target cell and stimulate protein biosynthesis. This would hasten regenerative processes and the recovery from the inhibition of mRNA formation in the intoxicated liver. From experimental and clinical experience, silibinin fulfills these characteristics.

## 3 THE DRUG LEGALON^®^ SIL

Legalon^®^ SIL is a fully developed and documented pharmaceutical formulation manufactured by Rottapharm / Madaus (Cologne, Germany). Its active ingredient is silibinin-C-2’,3-dihydrogen succinate, disodium salt Fig. (**1**). It has the molecular formula C_33_H_28_O_16_Na_2 _and a molecular mass of 726.56 g/mol. It is a microcrystalline powder that results from the esterification of silibinin with succinic anhydride to form its hydrosoluble disuccinic acid ester for parenteral application. Silibinin, a flavonolignan obtained by extraction of the seeds from milk thistle fruit (Ph. Eur.), is a mixture of two diastereomers, commonly named silibinin A and silibinin B. After the reaction of silibinin with succinic anhydride, silibinin-C-2’,3-dihydrogen succinate, disodium salt is obtained as a mixture of cis- and trans diastereomers of silibinin A and B dihydrogen succinates. Legalon^®^ SIL is currently registered and/or licensed in over a dozen European countries specifically for the treatment of *Amanita phalloides* intoxication.

Legalon^®^ SIL is formulated as a sterile lyophilisate in rubber-stoppered vials to be dissolved for intravenous infusion, containing 528.5 mg silibinin-C-2’,3-dihydrogen succinate, disodium salt which is equivalent to 350 mg of silibinin according to DNPH testing or 315 mg of silibinin according to HPLC determination. The recommended daily dose is 20 mg silibinin/kg via continuous infusion over 24 hours, following a single loading dose of 5 mg silibinin/kg on the first day of treatment. It is advantageous to begin treatment with Legalon^®^ SIL as early as possible, since survival has been correlated with the time of initiation of therapy [18]. However, in practical terms the onset of treatment is typically delayed up to 24 hours or more post presentation while the drug is being procured. Such delays do not appear to affect clinical outcomes provided that aggressive hydration has successfully prevented early renal failure.

### 3.1 Silibinin Pharmacology

From experimental results, silibinin strongly inhibits hepatocyte uptake of amatoxin by competitive inhibition of the same transporter system (OATP1B3) during both the primary and the enterohepatic circulation of the toxin [19, 20]. The latter mechanism is obviously relevant to human intoxication, since patients do not present until long after primary absorption has been completed. Based on current amatoxin toxicity knowledge, it is the period of time that the transcription process in hepatocytes remains blocked which is relevant for the prognosis. This period is largely determined by the amount of amatoxins taken up from the portal blood either primarily or during enterohepatic circulation [2]. A substance like silibinin that possibly can lower the amatoxin concentration at the target cell by blocking the transporter system used by amatoxin is therefore promising.

A second mechanism of action of silibinin has also emerged. Amatoxin appears to induce fulminant hepatic failure via TNF-α mediated hepatocyte apoptosis. It appears that silibinin may inhibit TNF-α release in the injured liver [21]. In addition, by stimulating protein synthesis silibinin can enhance the regenerative capacity of the liver. This is important because the blockage of mRNA transcriptional process by amatoxins leads to a lack of essential proteins after a latency period. By stimulation of rRNA synthesis silibinin increases the number of ribosomes in the cell and hence leads to an increase in biosynthetic capacity of the hepatocytes. Silibinin’s ability to improve the reduced protein synthesis rate should help prevent the development of liver and kidney failure [22].

For instance, a single oral dose of the lyophilized *Amanita phalloides* caused gastrointestinal signs of diarrhea, retching, and vomiting in beagle dogs after 16 hours. Liver damage occurred within 48 hours after poisoning and was evident by increases in serum transaminases (AST, ALT), alkaline phosphatase, bilirubin, prolongation of prothrombin time, and liver cell necrosis. Four of 12 dogs given the mushroom died with signs of hepatic coma within 35 to 54 hours and the biochemical values in the survivors reverted to normal by the ninth day. Intravenous silibinin (given as the water soluble succinate) at a dose of 50 mg/kg 5 and 24 hours after intoxication suppressed the serum changes and the fall in prothrombin time. The degree of hemorrhagic necrosis in the liver was markedly reduced in comparison to controls, and none of the dogs treated with silibinin died [23, 24]. Since dogs exhibit enterohepatic circulation of the toxins like humans, the dog model is considered appropriate in the study of human amatoxin intoxication. 

In summary, even though the precise mechanism of action of silibinin is still not totally elucidated, there is experimental evidence that silibinin may exert its hepatoprotective effects by

- Inhibition of the binding of amatoxin to hepatocyte membranes
- Competing with amatoxin for trans-membrane transport
- Interruption of biliary secretion and thus enterohepatic recirculation of amatoxin
- Inhibition of TNF-α release in damaged hepatocytes
- Stimulating protein synthesis in damaged liver cells.

There is also data suggesting that silibinin may work by decreasing the production of oxygen free radicals and lipidperoxidation that likely is part of the cell death process. It also seems to have an anti-inflammatory and antifibrotic effect [25, 26]. 

## 4 AMATOXIN POISONING IN HUMANS

Patients who consume amatoxin containing mushrooms exhibit symptoms and signs that typically occur in a progression of three clinical stages [9]. There is an initial quiescent period (6-24 hours post ingestion) after which, abdominal pain, nausea, vomiting, and severe watery diarrhea develop. It is at this point that most people seek medical care and may be misdiagnosed as having viral gastroenteritis. Routine laboratory values may reflect dehydration and electrolyte loss, but they are of little value in assessing the magnitude of intoxication or predicting the eventual outcome. Initial diagnosis and treatment of suspected amatoxin poisoning should not be delayed to wait for identification of uneaten mushrooms or detection of the toxin in body fluids.

The second stage of amatoxin poisoning is characterized by generalized clinical improvement (resolution of gastrointestinal symptoms) that begins 24 to 48 hours after ingestion, but masking the hepatic deterioration that is occurring at the same time. The liver function tests show a progressive elevation of transaminases and an evolving coagulopathy. Early renal damage may be reflected by elevations in the serum creatinine and blood urea nitrogen levels. 

Transition into the third stage can occur quite suddenly. Hepatic necrosis may be fulminant and is manifested by a severe increase in serum transaminases and a profound coagulopathy. Patients may experience a swift progression from stage I to stage III or IV hepatic encephalopathy. The presence of hypoglycemia and acidosis are poor prognostic signs reflecting massive hepatic necrosis. Renal failure, due to hepatorenal syndrome and/or direct nephrotoxicity of amatoxin, may result in severe oliguria or anuria. 

The outcome of amatoxin intoxication is linked to the stage of its progression when the treatment is initiated. The prognosis in untreated patients is as follows:

Grade 1: Patients develop gastroenteritis-like symptoms several hours (6-36) after ingestion but do not develop severe biochemical indications (peak transaminases <1,000 IU/L without coagulopathy) or renal dysfunction.

Grade 2: Patients manifest a moderate (1,000-5,000 IU/L) rise in transaminases and a mild (peak INR <2.0) (corresponding to Quick value of <37%) coagulopathy. 

Grade 3: Patients develop a marked elevation of transaminases (>5,000 IU/L) and significant coagulopathy (INR>2.0). Grade 3 is divided into two subgroups according to bilirubin values. In grade 3a bilirubinemia is mild or absent, while grade 3b shows a steep and continuous rise in bilirubin (greater than 5 mg/dL).

Grade 4: A steep rise in transaminases is accompanied by a corresponding steep decline in clotting function (INR>3.0) (corresponding to Quick value of >22%) and renal dysfunction. 

Grade 1 and grade 2 patients have the best chance of surviving amatoxin poisoning and need symptomatic treatment only. Grade 3 patients are at risk and should be transferred to a tertiary care center where liver transplant is available. Grade 4 patients have an extremely poor prognosis. There is a 90% probability of death in spite of intensive therapy. Survival usually requires a liver transplant.

## 5 CLINICAL EFFICACY IN *AMANITA PHALLOIDES* POISONING

Based on the above mentioned various experimental findings, a useful therapeutic approach for amatoxin poisoning would be to interrupt the enterohepatic circulation of the toxins and inhibit the trans-membranous transport into the cells thereby lowering the amatoxin concentration at the target cells [27].

Poisoned patients need fluid and electrolyte replacement, supportive measures to correct coagulation disorders, and measures to prevent the development of encephalopathy. Additionally, the use of other purported antidotes is common, although not significantly proven, mostly penicillin, N-acetylcysteine, or thioctic acid. The rationale for acute adjuvant treatment with penicillin or thioctic acid is mostly empirical. There is some evidence from cellular and animal models that penicillin also inhibits the transport system used by amatoxins [28]. In contrast, silibinin (Legalon^®^ SIL) has been shown *via* observational clinical trials and well documented case series to be an amatoxin antidote.

Phase III randomized controlled trials to prove the therapeutic significance of Legalon^®^ SIL are not possible due to the low incidence of amatoxin poisoning and the life threatening nature of the intoxication. This unique situation precludes the use of controlled trials versus placebo or other agents.

The time period between intoxication and initiation of treatment as well as the broad spectrum of necessary supportive measures can vary greatly between patients and makes structured clinical studies practically impossible. Therefore clinical observations during drug surveillance studies are considered as the only reasonable approach to collect data about the efficacy and tolerability of amatoxin poisoning antidote candidates.

### 5.1 Unpublished Clinical Data with Legalon^®^ SIL 

In an open multicenter clinical trial conducted in 1981 and 1982 in 6 European countries, 201 amatoxin poisoning victims received Legalon^®^ SIL in addition to penicillin (unpublished). Treatment effects by Legalon^®^ SIL were investigated in various subgroups, especially for ALT, AST, blood coagulation parameters and survival. Mortality dropped among all patients, regardless of the severity of intoxication, from more than 20% (historical data) to 10% when this cohort was compared to all cohorts prior to the use of Legalon^®^ SIL [29]. Therefore the use of Legalon^®^ SIL decreased mortality by 50% for this cohort.

Case reports for 154 amatoxin intoxicated patients treated with Legalon^®^ SIL between 1983 and 1992 were evaluated for efficacy and tolerability (unpublished). All patients had received Legalon^®^ SIL either as monotherapy (N=28) or in addition to penicillin (N=126). Course of intoxication and mortality rates were evaluated for both treatment regimens. Diagnosis was confirmed by gastrointestinal symptoms, clinical picture, and mushroom identification in most patients. The analysis of patient outcomes concluded that the overall mortality rate for the total 154 patients was 9.7%, confirming the above mentioned mortality reduction. Treatment with Legalon^®^ SIL was not only correlated with lower mortality but also with lower increases in transaminases and a less severe coagulopathy. There was only one death (4%) in the Legalon^®^ SIL monotherapy group, while 14 patients died in the Legalon^®^ SIL plus penicillin group (11%).

### 5.2 Published Clinical Experience with Legalon^®^ SIL 

Between 1982 and 2008, mostly single cases were published by various authors (Table **2**). Overall, out of these 1,136 reported patients treated with Legalon^®^ SIL as monotherapy or in combination with mostly penicillin G, 1,064 patients (94%) survived. It was further reported that the severity of liver damage correlated with the time interval between mushroom intake and commencement of silibinin treatment. If silibinin was given within 48 hours after intoxication, a light or medium hepatic injury was expected. When silibinin treatment was delayed by more than 48 hours, then a severe clinical course with coagulation disorders and liver coma was more likely [30]. 

In a 20-year retrospective analysis published in 2002, clinical data from 2,108 hospitalized amatoxin-intoxicated patients from North America and Europe was analyzed to measure the effects of various antidotes. No significant therapeutic effect was observed for thioctic acid, steroids, or penicillin G. Silibinin as Legalon^®^ SIL and N-acetylcysteine showed the most positive effects. Of the 624 intoxicated patients treated with Legalon^®^ SIL, 589 (94%) survived [28].

In 2008, a comparison of Legalon^®^ SIL monotherapy versus combination therapy of Legalon^®^ SIL and penicillin in 367 case reports of *Amanita*-intoxicated patients from 92 hospitals in 11 countries was published [31]. One hundred and eighteen patients received Legalon^®^ SIL alone and 249 patients received the silibinin infusion therapy in combination with penicillin. Regression analyses showed a lower combined mortality and transplantation rate of 5.1% with Legalon^®^ SIL monotherapy compared to 8.8% with the combination treatment.

The first use of silibinin as Legalon^®^ SIL in an American cohort was reported in 2008 [32]. An immigrant Mexican family of 6 ate tacos containing *Amanita phalloides*. Symptoms began 8 hours later with hospital presentation approximately 15 hours post ingestion. All received aggressive intravenous hydration, intravenous penicillin, and N-acetylcysteine. By 72 hours post-ingestion, 4 had developed laboratory evidence of fulminant hepatic failure.

The family was transferred to a liver transplant program in San Francisco. An Emergency Investigational New Drug application for Legalon^®^ SIL was granted by the FDA, and the drug was couriered to California. Infusions of 5 mg/kg every 4 hours were initiated in all 6 patients beginning about 78 hours post ingestion. Two of the 4 family members with fulminant hepatic failure recovered rapidly and were discharged after 5 days of silibinin therapy. The sickest member of the family was a 29 years old male with a peak INR of > 15 who was listed for transplant. But he fully recovered without liver transplantation and was discharged 11 days after mushroom poisoning. An 83-year-old woman ultimately succumbed to anuric renal failure, although her liver appeared to have recovered significantly within 48 hours after silibinin treatment was begun. All survivors had completely normal laboratory values 2 months later. 

Overall, taking together the available clinical data (unpublished and published), from a total of 1,491 documented *Amanita*-poisoned patients treated with Legalon^®^ SIL 1,384 survived, resulting in a survival rate of 93% (Table **2**). 

## 6 SAFETY 

Legalon^®^ SIL is generally well tolerated. From drug surveillance and periodic safety updates over the years since introduction of the product in 1984, no serious adverse drug reactions have been reported. Flushing during the intravenous treatment is not uncommonly reported but appears to be of mild degree. From approx. 9,000 patients, as estimated by product sales, Legalon^®^ SIL infusion therapy can be considered as safe.

## CONCLUSIONS

Human intoxication with *Amanita phalloides* and related species is associated with severe morbidity and a high mortality rate due to progressive fulminant hepatic failure. For ethical and practical reasons there are no controlled clinical studies available for any amatoxin treatments but observational studies and case reports describe successful treatment with intravenous silibinin (Legalon^®^ SIL). 

There is experimental evidence for silibinin’s mechanism of inhibiting amatoxin uptake into hepatocytes as well as biliary secretion due to the blockade of membrane transporter systems. The pronounced antioxidative properties of silibinin may play an additional beneficial role in decreasing oxidative stress in the damaged liver. Given the similarity to human intoxication pathology, the reduction of liver cell damage measured by laboratory parameters, histology and mortality rates after intravenous application of silibinin in a pharmacological dog model is noteworthy. 

There are currently more than 1,300 documented cases supporting the clinical efficacy of silibinin-based Legalon^®^ SIL as an antidote in patients with acute amatoxin poisoning. This represents the largest patient population ever analyzed for this potentially fatal intoxication. The overall mortality rate for Legalon^®^ SIL treated patients regardless of the severity of liver damage is less than 10%. This represents more than a 50% reduction in the reported mortality rate before Legalon^®^ SIL was available. Legalon^®^ SIL can be used safely with a positive benefit / risk ratio. 

## Figures and Tables

**Fig. (1):**
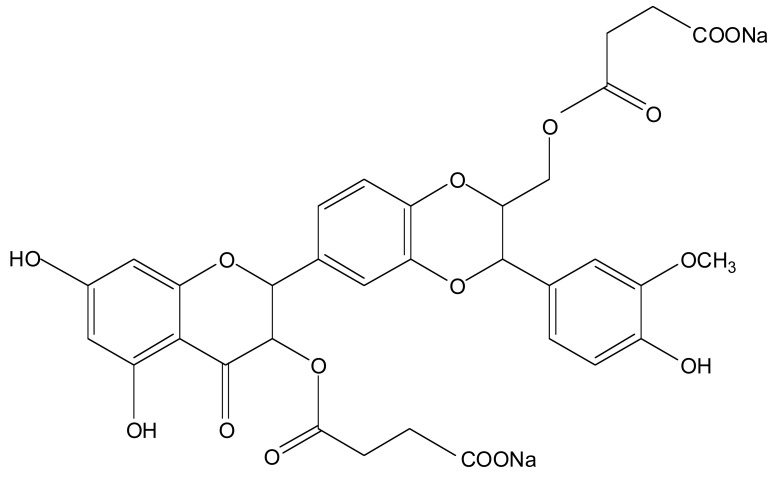
Silibinin-C-2’,3-dihydrogen succinate, disodium salt.

**Table 1.** Toxic *Amanita* Species

*Amanita* Species	Common Name
*Amanita phalloides*	Deadly *Amanita* Death angel Death cap
*Amanita verna*	Spring Amanita White death cap
*Amanita virosa*	White *Amanita* destroying angel

**Table 2.** Summary of Clinical Experience with Legalon® SIL in Amatoxin Poisoning

Reference	Treatment Regimen	No. of Patients	
		Treated	Survived
Lorenz *et al.*, 1983 (unpublished)	PE + SIL	201	181
Strenge-Hesse *et al.*, 1996 (unpublished)	PE + SIL	154	139
Floersheim *et al.*, 1982 [29]	PE + SIL	16	16
Hruby 1983 [30]	PE + SIL	15	14
Marugg and Reutter 1985 [33]	PE + SIL	12	11
Smetana *et al.*, 1986 [34]	PE + SIL	2	2
Schenke *et al.*, 1987 [35]	SIL	2	2
Hruby 1987 [36]	SIL PE + SIL TH + SIL PE + TH + SIL	17 37 1 15	16 34 1 15
Kelbel and Weilemann 1989 [37]	PE + SIL	5	4
Nagy *et al.*, 1994 [38]	PE + SIL	4	3
Kleist-Retzow *et al.*, 1995 [39]	PE + SIL	2	2
Molling *et al.*, 1995 [40]	PE + SIL	2	2
Carducci *et al.*, 1996 [41]	PE + SIL	4	4
Alves *et al.*, 2001 [42]	PE + SIL	4	4
Boyer *et al.*, 2001 [43]	NAC + SIL	1	1
Enjalbert *et al.*, 2002 [28]	SIL or PE+SIL	624	589
Ganzert *et al.*, 2008 [31]	SIL or PE+SIL	367	339
Mitchell and Olson 2008 [32]	PE+NAC+SIL	6	5
Total		1,491	1,384

PE=penicillin, SIL=Legalon® SIL, NAC=N-acetylcysteine, TH=thioctic acid.
